# Health inequalities as a foundation for embodying knowledge within public health teaching: a qualitative study

**DOI:** 10.1186/1475-9276-12-46

**Published:** 2013-06-28

**Authors:** Mzwandile A Mabhala

**Affiliations:** 1 Department of Community Health and Wellbeing, University of Chester, Riverside Campus, Chester CH1 1SF, UK

**Keywords:** Social Justice, Inequalities in Health, Public Health, Embodying Knowledge

## Abstract

**Introduction:**

Recent UK health policies identified nurses as key contributors to the social justice agenda of reducing health inequalities, on the assumption that all nurses understand and wish to contribute to public health. Following this policy shift, public health content within pre-registration nursing curricula increased. However, public health nurse educators (PHNEs) had various backgrounds, and some had limited formal public health training, or involvement in or understanding of policy required to contribute effectively to it. Their knowledge of this subject, their understanding and interpretation of how it could be taught, was not fully understood.

**Methodology:**

This research aimed to understand how public health nurse educators’ professional knowledge could be conceptualised and to develop a substantive theory of their knowledge of teaching public health, using a qualitative data analysis approach. Qualitative in-depth semi-structured interviews (n=26) were conducted with eleven university-based PHNEs.

**Results:**

Integrating public health into all aspects of life was seen as central to the knowing and teaching of public health; this was conceptualised as ‘embodying knowledge’. Participants identified the meaning of embodying knowledge for teaching public health as: (a) possessing a wider vision of health; (b) reflecting and learning from experience; and (c) engaging in appropriate pedagogical practices.

**Conclusion:**

The concept of public health can mean different things to different people. The variations of meaning ascribed to public health reflect the various backgrounds from which the public health workforce is drawn. The analysis indicates that PHNEs are embodying knowledge for teaching through critical pedagogy, which involves them engaging in transformative, interpretive and integrative processes to refashion public health concepts; this requires PHNEs who possess a vision of what to teach, know how to teach, and are able to learn from experience. Their vision of public health is influenced by social justice principles in that health inequalities, socioeconomic determinants of health, epidemiology, and policy and politics are seen as essential areas of the public health curriculum. They believe in forms of teaching that achieve social transformation at individual, behavioural and societal levels, while also enabling learners to recognise their capacity to effect change.

## Introduction

The health policies of successive UK governments put emphasis on public health as a strategy to tackle inequalities in health [1-10], and nurses, health visitors and midwives were identified as key contributors in tackling them [11-14]. However, several public health nurse researchers have argued that for nurses to contribute to tackling inequalities in health, they must first be educated on how social inequalities are created and sustained [15-17].

In response to this challenge, there has been an increase in public health content within pre-registration nursing education programmes [18-21], developed and taught by nurse educators from highly contrasting backgrounds. Although most nurse educators have formal schooling in nursing and teaching, most have had no formal schooling in the principles and practice of public health; nevertheless some have a specific remit to teach public health.

It has been argued that higher education teaching requires synergy of knowledge of subject concepts and content, and knowledge of how to make that content intelligible to learners [22-27]. However, whilst there have been numerous initiatives to develop public health knowledge and skills for public health specialists and practitioners, the evidence from a number of reports is that public health professionals come from a wide variety of backgrounds and possess varying levels of knowledge and skill [13,28,29]. No research has considered the implications of these differences for the practice of public health teaching.

Most of the published public health reports lack specificity about the conceptual meaning of possessing knowledge and skills in public health. They focus narrowly on factual evidence about the type of formal educational qualification (‘knows what’) and experience in public health (‘knows how’) [13,28,29]. This appears to be based on the assumption that individuals presenting this factual evidence have the necessary public health principles, values and beliefs. Not many reports considered the variation in people’s embodied knowledge [30] of public health. Such variation raises significant questions: What are public health nurses supposed to know in order to contribute meaningfully to strategies to reduce inequalities in health? What does knowing public health mean? In spite of this evidence not much is published about PHNEs’ understandings and interpretations of public health, or of public health interventions to reduce inequalities in health. The researcher has been in public health practice for 18 years, in middle and high income countries; and has had opportunity to use these experiences to teach public health. During this time has observed that there were variations of interpretations PHNEs attached to the public health concepts taught within the nursing curriculum, which created a need to develop a research-based explanation for these variations.

The purpose of this research was to provide an explanation of how PHNEs’ professional knowledge could be conceptualised and to develop a substantive theory of their knowledge of teaching public health. The ultimate purpose of this research is to improve the training of PHNEs by identifying gaps in PHNE’s knowledge and experience that need to be filled, and thereby improve the teaching of public health.

## Study design and methods

The design of this study was influenced by Charmaz’s [31,32] constructivist grounded theory. The stages of data collection and analysis drew heavily on other variants of grounded theory, including those of Glaser and Strauss [33], Strauss and Corbin [34,35], and Charmaz [31,32]. Although constructivist grounded theory has been criticised as not being compatible with classic grounded theory [36,37], it has been used here because it closely matched the theory-seeking nature and paradigm that underpinned the conduct of this study.

One aspect of this research consistent with constructivist grounded theory was its fundamental ontological assumption of multiple realities [31,32], constructed through the experience and understanding of different participants’ perspectives, and generated from their different academic, social, cultural and political backgrounds. Another was its epistemological belief that public health knowledge is shaped by the cultural, historical, political and social norms that operate within that context and time. These assumptions outlined the importance of taking account of the influence of the researcher’s involvement, and the influence of the contexts that surrounded data collection both in time and locality [31].

### Recruitment of participants and setting

The setting for this study was the Faculty of Health and Social Care in one of the English universities. The participants were selected from the population of 98.6 full-time equivalent nurse educators in the faculty. The participating institution delivers an undergraduate nursing curriculum in five campuses. Within it there is a public health module, with designated public health module leaders and deputies on each campus. The investigator was one of eight public health module leaders at the time of this investigation.

Three sampling strategies were used in this study: purposive, criterion and theoretical. It started with purposive sampling and in-depth one-to-one semi-structured interviews with six public health module leaders from four of the five campuses. These were invited directly by the investigator. The purpose of this initial sampling was to generate themes for further exploration.

One of the main considerations for the criterion recruitment strategy was to ensure that the process complied with the ethical principles of voluntary participation and equal opportunity to participate. To achieve this; an email was sent to all nurse educators within the faculty inviting them to participate. Within the email the purpose, nature of the study and the type of data the study aimed to elicit were made explicit. To help potential participants make a self-assessment of their suitability to participate without unfairly depriving others of the opportunity, the email explained that potential participants should meet at least one of the following criteria: currently teach or have taught public health; currently work or have previously worked in a public health related field; have a special interest in public health; and see the specific relevance of public health to their area of practice. Eighteen participants met the criteria for inclusion and sixteen agreed to participate. After excluding previously interviewed six module leaders; eleven were actually interviewed for this phase of investigation. All eleven participants had taught public health and/or health promotion and nine of them were current public health teachers.

As categories emerged from the data analysis, theoretical sampling was used to refine undeveloped categories. Theoretical sampling was undertaken in accordance with Strauss and Corbin’s [34] recommendation that the filling in of poorly developed categories be done through review of memos or raw data, looking for data that might have been overlooked [38]; and returning to key participants asking them to give more information on categories that seemed central to the emerging theory [39,40]. A total of 26 interviews were conducted with the eleven participants including one of the six module leaders from the initial sample. The process involved iteration between analysis and data collection; this meant that the investigator had to determine the sources of data and/or which participants were likely to provide the rich data needed for category development [39]. The questions asked at this stage were guided by the analysis; these included questions such as:

Possessing a wider vision of public health

1. In your earlier interview you mentioned that that your perception of public health is influenced by your personal life. Could you elaborate on how your personal life including family, community, social life, schooling and work experiences influence your understanding and interpretation of public health?

2. Could you talk about incidents in your professional life that explain your perception of public’s health?

Engaging in appropriate pedagogical practices

1. How are your experiences, values and perceptions of public health reflected in your teaching?

2. How much of what you teach is informed by your personal experience, and how much by formal public health learning?

3. How much of your teaching style is influenced by your understanding of public health content?

4. How much of your teaching style is influenced by the way you learned public health?

### Data collection

Semi-structured interviews were used to collect data. Each interview was 60 to 90 minutes in length. Interviews were audiotaped and transcribed by the investigator. Data collection took place between April 2006 and February 2010, and ‘theoretical sufficiency’ ([41], p. 117) was achieved after a total of 26 qualitative interviews had been conducted. This study adopted the same meaning of theoretical sufficiency as Díaz Andrade [41] which is ‘that categories have been developed to a sufficient extent, so that it is possible to explore their relationships and draw some conclusions’ ([41], p. 48). Some would describe this as theoretical saturation [34]; however in this study the term “theoretical sufficient” was considered more appropriate than theoretical saturation. While both indicate that the data have been properly analysed; theoretical sufficiency was preferred instead of “theoretical saturation” as the former, acknowledges that the process of generating categories can never be absolutely exhaustive [41,42].

### Data analysis

**Figure 1:**
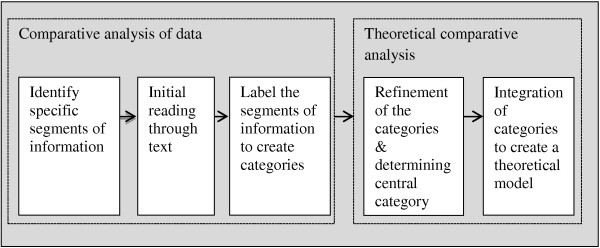
Summary of QDA process of data analysis and theory building.

In this study data collection and analysis occurred simultaneously. Analysis drew on the grounded theory principles of constant comparative analysis and the iterative process of data collection and data analysis to build theory inductively. The data analysis was broadly organised according to the two phases of comparative analysis – making a constant comparative analysis and making a theoretical comparison [38] – a process summarised in Figure 1.

At the comparative analysis phase (Figure 1), each interview was read line-by-line to identify segments of data that contained theoretically significant incidents: incidents in the data that appeared to have potential to render a theoretical explanation of the core phenomenon under investigation.

The theoretically significant incidents were coded in accordance with Strauss and Corbin’s [34] open coding, which is defined as an ‘analytic process through which concepts are identified and their properties and dimensions are discovered in data’ ([34], p. 101).

As data collection and analysis progressed, each incident in the data was compared with incidents from both the same participant and other participants, looking for similarities and differences [34,38]. Significant incidents were coded or given labels that represented what they stood for [38], and coded or given the same labels when they were judged to be about the same topic, theme or concept [38]. After a period of interrogation of the data, it was decided that the three categories – possessing a wider vision, reflecting and learning from experience, and engaging in appropriate pedagogical practices – were sufficiently conceptual to be used as theoretical categories around which subcategories could be grouped.

Once the major categories had been developed, the next step consisted of a combination of theoretical comparison and theoretical sampling. The emerging categories were theoretically compared with the existing literature [38]. Once this was achieved, the next step was filling in and refining the poorly defined categories. This process continued until theoretical sufficiency was achieved.

### Ethical considerations

Ethical approval was obtained from the University Ethics Committee after their review of the study design, tool used and other research material, and of the participant information sheet which included a letter of invitation highlighting that participation was voluntary.

To comply with the principles of voluntary and informed consent, potential participants were provided with information about the study (aims, objectives, and the voluntary nature of participation), and were then invited to take part [43,44].

Confidentiality of data and anonymity of participants were assured with all identifying information removed from transcripts, data stored in a secure location, data reported in aggregate form and data accessible to researchers only [43].

## Results

The research set out to determine how public health nurse educators’ knowledge of teaching public health is conceptualised. The data in this study revealed that their knowledge could be conceptualised as ‘embodying knowledge’. The concept of ‘embodying knowledge’ seemed to fit the data, and offered one interpretation of the practice of knowing and teaching public health. Other categories fitted logically with the central category. The breadth and depth of categories and properties appeared to explain what the research was about.

**Figure 2:**
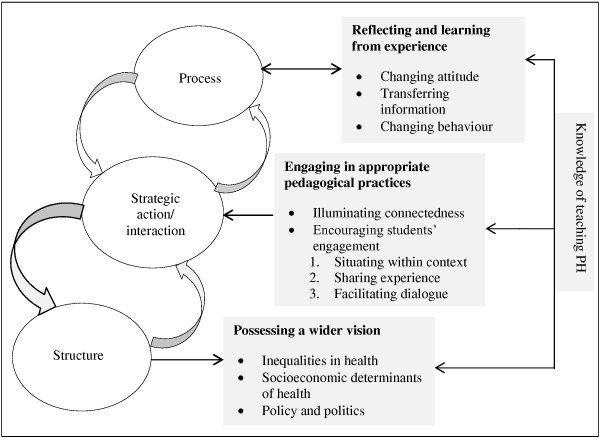
Model of embodying knowledge for teaching public health.

Like any category the central category needed to be defined in terms of its properties. In this case, although the concept of embodying knowledge was not used in the interviews, the memos were replete with references to ‘integrating public health into all aspects of life’, ‘making public health part of everyday life’, and ‘fully embracing public health’ which are all properties of ‘embodying knowledge’. Therefore, ‘embodying knowledge’ within this study was defined as the process of reflecting and learning from various experiences in which people engage, in order to make sense of the received information and interpret it according to their personal and professional relationship with the subject: taking ownership of the subject and integrating it into everyday life. The conceptual categories that provide theoretical explanation to the central category are: (a) possessing a wider vision of health; (b) reflecting and learning from experience; and (c) engaging in appropriate pedagogical practices. Figure 2 illustrates the interrelationship among three elements of embodying knowledge for teaching public health.

### *Possessing a wider vision of public health*

PHNEs who are embodying knowledge for teaching public health possess a vision of public health being an integral part of all facets of an individual’s life. Their understanding of public health concepts reflects that wide range of experiences upon which their knowledge was drawn. They believe that social justice is a foundation for public health and in using it as a strategy to reduce inequalities in health. In this study social justice refers to the idea of creating a society or institution based on the principles of equality and solidarity, that understands and values human rights, and that recognises the dignity of every human being.

Themes considered consistent with social justice, and thus essential components of the structure of the public health curriculum, are: (a) epidemiology of health inequalities; (b) socioeconomic determinants of health (SEDH); and (c) engaging with policy and politics. Of the three, understanding the epidemiology of health inequalities was considered the foundation on which the whole public health curriculum was built. This statement by one of the public health nurse educators from a district nursing background, who holds a Master of public health qualification, represents the views of all the participants in this study:

*‘Health inequalities is the main thing, epidemiology is another one, it’s identifying where health issues are, where they come from and what the causes of them are and you can only do that by studying the population group looking at the epidemiology of the population, identifying the strategies perhaps to try and prevent ill health by working upstream by sort of putting together preventive strategies and I think in specialist practice programme with variety of students some do work in the primary prevention and other students and I think district nurses work with clients groups who have after-effects of years of ill health.’* [PHNE4]

However, PHNEs’ interpretations of these public health concepts varied according to their personal and professional backgrounds. These differences related to ideological positions on how inequalities in health might be tackled. Some believed that this requires advocacy and empowerment of vulnerable individuals to change behaviour and adopt healthy lifestyles. One of the comments that illustrate this point was from nurse educators from a health visiting background:

*‘Public health is an intervention to change behaviour. I think that if they have a grasp of what inequalities in health might be, I’m hoping that they might be able to identify individuals as well as groups that fit into those categories, subsequent when it comes to them having to give actually information or being on board with any kind of advice, they have some kind of understanding of why people behave the way they do. It’s very much on the individualistic basis underpinned by some of the theories that we’re actually trying to give them.’* [PHNE3]

This idea of tackling inequalities in health was consistent with one by a public health educator from a school nursing background, who holds a Master of Public Health degree. She stated that:

‘I think health is a personal choice, but is a choice with caveat. You can only make choice if you are informed and also if are in that arena in the circle of change that actually enables you to make that change you need to make that where advocacy comes in. We all know from our experiences for example, smoking cessation that people fail several times and failure reinforces that belief that they cannot achieve the cessation position but in actual fact with advocacy and support and information we can move people to that point.

*I think advocacy is very important because all people are vulnerable at some stage in their lives, some people move into the stage of vulnerability and move out of it very quickly and others live in almost permanent state of vulnerability. And it is the vulnerable that suffer the greatest inequalities in health and therefore if we lose vision to advocacy, we lose power to help these people to move on from that state of vulnerability.’* [PHNE8]

Others believed that inequalities were created and sustained by unfair social policies and social institutions; and therefore that the solution lies with upstream transformation of systems that create and sustain social and economic inequalities. Among the few participants who expressed this view was a nurse educator from a general nursing background who holds a MSc in Health Promotion:

*‘Student nurses are citizens of the country and also nurses. I think you have to engage with policy and politics because otherwise from the health point of view I don’t think we are going to see any great changes, because any changes have to be supported by the government. We can educate people about health but unless inequalities on things such as education, environmental issues, if they aren’t addressed, then we will have a very limited impact.’* [PHNE2]

### *Reflecting and learning from experience*

Reflecting and learning from personal and professional experiences were central to embodying knowledge for teaching public health. These experiences are conceptualised in this study as ‘intellectual biographies’ – the set of understandings, values, beliefs, experiences, conceptions and orientations that constitute the source of participants’ comprehension of the subject. The three processes through which PHNEs embodied knowledge for teaching public health were received information, attitudinal change, and behaviour or practice experience.

**Figure 3:**
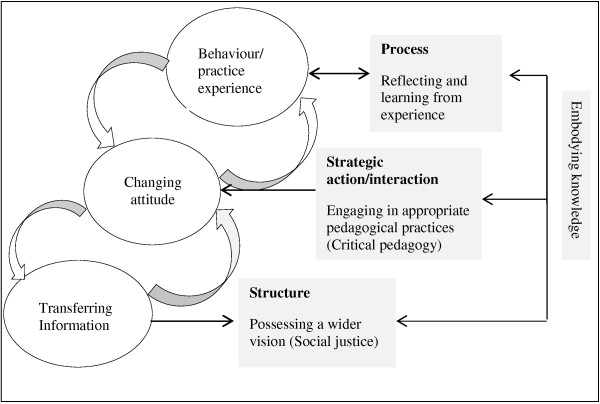
Acquiring and integrating public health knowledge.

Figure 3 shows a complex implicit link between information, attitude and behaviour. Distinguishing/identifying the first and last piece of the three piece chain was complex. For some, the chain seemed to begin with attitudinal change which created a need for acquisition of information, evidence-based data to support their beliefs; in this case one could argue that if you could sufficiently change people’s feelings towards the subject, they would want to integrate it into their everyday life. For others, the chain began with people doing things, and reflection on their action over time resulted in integrating values and beliefs into their everyday lives.

Participants in this study proposed that how people feel (attitude) about the connection between public health and one’s areas of interest was a necessary condition for the integration of public health into one’s everyday practice. This was made explicit by a participant from a mental health background. This participant has neither direct experience nor qualification in public health. He proposed that selection of who teaches public health should be based on ability to accurately interpret the connection between inequalities in health and poor health, rather than availability to teach:

‘Forgive my cynicism, but it appears to me that the decision in my department [mental health] about who teaches public health is based on availability rather than ability to see the connection between public health and mental health. Don’t get me wrong, I don’t mind stepping in.

*I wouldn’t say I necessarily have public health expertise, but I see the connection between public health and mental health, I always believe that the pattern of disease distribution follows the pattern of how privileges are distributed, I have lived and worked in North Wales and Northwest of England long enough to know that and believe that one way of improving health of many people in our society is to tackle inequalities in health.’* [PHNE11]

Another nurse educator, from a surgical nursing background, explained the relevance of public health in the surgical nursing speciality as:

*‘I came from the surgical background. I can see the relevancy of public health in surgical speciality, because it affects the whole well-being of our patients. Although I don’t do much of public health teaching any more but I am still interested in public health as it affects my patients. You can see influence of inequalities in health even in surgical wards that I think as a nurse I have a moral obligation to advocate for my patients against inequalities in health.’* [PHNE10]

The ability to see the connection between public health and professional practice was also expressed by a PHNE with a learning disability background who explained that:

‘Public health probably relates more strongly to LD now than it has done in the past. There is a growing awareness of inequalities in health particularly in relation to people with the learning disabilities and the role of the learning disability nurse has become very much health oriented particularly in the last few years. So the link now is probably stronger now than has been for quite few years.

*Obviously I am more interested in it as it relates to people with learning disability so areas such as inequalities in health that sort of thing and also general health policies and how that gets related to people with learning disabilities.’* [PHNE7]

One of the processes by which nurse educators in this study acquired public health knowledge was information transfer. This involved people encountering situations in their personal or professional lives that challenged their existing knowledge, and then deciding to undertake an academic course to fill the gaps in their knowledge:

*‘As an occupational nurse I had an interest in relationships between work and health. I read a report into pensionable ages of the prison civil service staff. It reported that the average prison officer on retirement only lives for six months post retirement and die usually from coronary heart disease (CHD). This further stimulated by awareness that people in civil service such as nursing don’t really live to enjoy the pension that really stimulated my interest in how work influences your life. I was just fascinated by how the determinants of health influence us really as human being. I decided to go and do master’s in public health.’* [PHNE1]

From this and other examples it was clear that some participants already knew most of the public health concepts at the time of undertaking an academic programme; the information gained from doing the MPH (Master of Public Health), which included epidemiology and inequalities in health, gave them the context and background evidence to support what they already knew about public health. This statement represents the view of three participants who hold master’s degree in public health in this study:

*‘When I came to [teaching] I needed to have master’s degree in order to be given a permanent contract….. So I went to do MPH. Although I knew about the issues of inequalities in health from when I was working in district nursing because the area that I work geographically was an area of high deprivation. Doing MPH, all the data, all epidemiology that give you the context, gives the background evidence to support all that really.’* [PHNE4]

This PHNE’s comments, like those of most participants in this category, reflect the complexity of the relationship between processes – changing attitudes, transferring information and changing behaviour – by which nurse educators in this study acquired and embodied public health knowledge. Even though this participant holds a MPH qualification, and has several years of district nursing experience and a senior lecturing position, she considered a lack of public health practice experience as a significant gap in her public health expertise. She explained that her lack of practical public health experience meant that her teaching was purely theory based; there was no practical experience to draw upon.

*‘The only problem is that I find to be honest is that I have never had an opportunity to work in a public health arena, all my knowledge is theoretical, because I was taught theoretical. I did a theoretical programme of study and now I teach the theoretic element of it, I have never actually worked in public health arena although my background is community nursing. That is something I would like to do, to go out and spend time with them, I have tried to do, but they were so short staffed, I wanted to shadow them to see what they did so then I can come back to talk to the students with more practical application.’* [PHNE4]

The features that emerged in this nurse educator’s comment, namely ‘use of theoretical evidence’ and ‘access to formal education’ to inform practice experience, were also evident in other participants’ comments.

The third process by which PHNEs acquired public health knowledge is behaviour or practice experience; that is, knowledge gain that occurred through people actually doing something. For example, as nurses were working in various healthcare settings they were continually reflecting on the connectedness of public health concepts to what they were doing, and over time this became an integral part of their practice. As one PHNE asserted:

*‘It [public health] sort of comes natural for me because public health and community nursing are a big part of my nursing care role, and then that was sort of formalised with revalidation of specialist nursing programme. With the change in national health policy, occupational health nursing is now a specialist public health route. This has provided more structure within the public health agenda.’* [PHNE6]

### *Engaging in appropriate pedagogical practices*

PHNEs in this study were engaged in pedagogical practices aimed at illuminating the connectedness between public health concepts and everyday lives. They expressed concerns about public health being treated as a separate entity from other aspects of nursing professional practice. This concern about the fragmentation of their subject and the lack of consolidation represented the feelings of PHNEs across all branches of nursing. It was therefore suggested that teaching strategies need to consider ways to fit together the jigsaw pieces that make up public health:

*‘Public health is seen as a separate entity as everything is seen as a separate entity, but none of them is a separate entity – they are all holistic, they are all combined so when you start off from day one public health should be there and should go through to the end, but it doesn’t at the moment; there is a block here and block there, we have no mechanism to pull all the jigsaws so that they can see where everything fits.’* [PHNE5]

They proposed pedagogical approaches that specifically emphasise the importance of making it possible for learners to see the inextricable connection between public health concepts, personal and professional lives, and the broader societal context in which health inequalities are generated; and of developing an intimate understanding of the social orders, processes and practices that sustain and mask social injustices.

*‘I am a big fan of case scenarios, giving students complex cases to look at and again sometimes you can see those wonderful public health principles sort of fitted into sort of like complex family agendas with so many different facets that are affecting health. I think a good way will always be problem-based learning, encouraging group discussion, giving groups case scenarios to work through often of complex public health issues, lifestyle issues… perhaps overweight, alcohol, no job at all, living in socially deprived area, get students to think about all those different facts really as opposed to generally standing there and telling them that is what public health is. Moving that into them looking at complex cases, I think it mirrors life more effectively. Get them thinking, really, about how they can work differently, and also how they can work collaboratively, what other services that are involved in a situation.’* [PHNE1]

## Discussion of findings

In this study, inequalities in health, socioeconomic determinants of health (SEDH), policy and politics, and epidemiology were identified as key components of the public health curriculum. Possessing a wider vision of public health was considered as being able to see the connectedness between public health and the wider socioeconomic and political systems that produce and sustain inequalities in health. This was consistent with WHO’s understanding of the public health function: creating the social, economic, political, cultural and environmental conditions necessary for all people to lead healthy lives [45-47]. However, this study revealed that the challenges facing nursing education included variations in the extent of PHNEs’ understanding and integration of public health principles into their everyday practices. The variations about their construction of meaning around public health and health inequalities were evident from the two specialist community public health nurses (PHNE 4 and PHNE 8) who understood public health as the context for improving individual behaviour. They understood interventions to reduce the inequalities as enabling people to make healthy choices by means of advocacy, empowerment, information and support. While nine other participants understood inequalities in health as created by unfair and unjust policies and practices that preferentially reward certain groups, economically and socially, at the expense of others; and tackling the inequalities in health as addressing upstream, socioeconomic determinants of health inequalities.

These variations amongst PHNEs were also reported in studies that investigated the role of public health nurses in reducing inequalities in health practices; these concluded that, although some nurses contribute to the public health agenda, there were significant differences between nursing disciplines in relation to the extent of activity within specific areas of public health [48-51]. In the UK it has been found that, contrary to the governments’ endorsement of their public health role, nurses spend a substantial proportion (61%) of their time on intervention at individual levels [48-50,52].

The second challenge is seeing the connectedness of public health concepts to their nursing profession, as well as to other, wider social contexts. This study revealed that the processes by which PHNEs engaged with public health concepts and integrated them into their everyday practice are transferring information, changing attitudes and changing behaviour. The theoretical model of embodying knowledge in Figure 3 illustrates the complex interaction amongst these processes. This study found that embodying knowledge through integration of received information with personal, professional and organisational values and beliefs is an indication of reflecting and learning from experience. This capacity to learn from one’s own and others’ experiences through active reflection has been reported as one of the essential qualities of the critical pedagogical approach to teaching [27,51,53]. As Shulman and Shulman [27] explain ‘if an [educator] were merely capable of vision, motivation, understanding, and practice, he or she would still lack the capacity for learning from experience and, thus, the capacity for purposeful change’ ([27], p. 264). These findings are also comparable to Freire’s [53] theory that the more reflective of our own experiences we are, the more conscious we are of our understandings and need for further learning.

The new perspective that emerged in this study and was not included in previous reports, such as *Chief Medical Officer’s Report on public health workforce*[13], *How and what teachers learn: A shifting perspective*[27], and *Public health skills and career framework*[28], was articulating the influence these varying experiences have on public health professionals’ understanding and conception of public health as a strategy to reduce inequalities in health.

It emerged in this study that PHNEs’ pedagogical approaches aimed to address the challenges relating to nurses’ understanding of public health reflect similarities with the values of Freire’s [53] critical pedagogy. Zimmerman et al. [51] defined critical pedagogy as pedagogy based on critical theory, a movement which seeks to analyse oppressive practices that lead to social inequalities experienced by members of society, especially those who are marginalised [51].

As this and several other studies [54,55] confirmed, critical pedagogy in the context of public health and social justice enables educators to illuminate connectedness, encourage students’ engagement with public health concepts, situate public health within context, share experiences and facilitate dialogue whilst sensitising the students to injustice, inequality and domination, issues relevant to all health contexts [54-59]. This and other studies found that critical pedagogical approaches increase ethical consciousness in both students and nurse educators [54]. For example, Lynam et al. [59] and Lynam [58] found that educators who base their teaching on critical pedagogy analyse social life through the lens of diversity and social justice, and prepare students to be transformative democratic agents. Their teaching strategies involve recognising and taking into account the broader societal context; its impact on local problems and social change are articulated. Consistent with Chávez, Turalba and Malik [56], PHNEs reported that the significant tenet of their pedagogical approaches was to create a balanced environment for learning public health by facilitating critical dialogue about social conditions, giving students a voice, and motivating them to reflect on their lives and take action.

## Conclusion

PHNEs envisioned tackling health inequalities as being a foundation on which public health curricula should be built, and identified SEDH, policy and politics as key components. These findings, together with data from the literature review, led to the conclusion that social justice was the underpinning principle behind participants’ public health vision. It emerged in this study that embodying social justice requires nurses who possess a wider vision of public health – the ability to see the connectedness between public health and the wider socioeconomic and political systems that produce and sustain inequalities in health. However, this study revealed that there are variations in understanding or embracing social justice principles amongst nurses. These findings remind us that public health knowledge is context situated; therefore, when making decisions about people’s public health knowledge and skills, the public health community needs to consider the context within which that knowledge was developed and used.

### Limitations

Because participants were recruited from one faculty in a relatively mid-size university, one limitation of this study related to the representativeness of the sample. Public health educators (academics) come from wide range of backgrounds; the sample in this study consisted of one group of public health educators (nurses), and in a larger population that included other disciplines the results might be different. The investigator was known to some of the participants; though every effort was made to account for respondents’ bias, this can be seen as a potential limitation of this study. It has to be acknowledged that the method of recruitment of the 11 participants generates a bias in favour of those with a particular interest in public health. The methodology used in this study (constructivist grounded theory) advocates mutual construction of knowledge that means researcher’s understanding and interpretations may have had some influence in the research process as the researcher is an integral part of the data collection and analysis.

## Competing interests

The author declares that he has no competing interest.
